# The functional anatomy of attention: a DCM study

**DOI:** 10.3389/fnhum.2013.00784

**Published:** 2013-12-02

**Authors:** Harriet R. Brown, Karl J. Friston

**Affiliations:** The Wellcome Trust Centre for Neuroimaging, Institute of Neurology, University College LondonLondon, UK

**Keywords:** attention, active inference, predictive coding, precision, Posner, cortical gain control

## Abstract

Recent formulations of attention—in terms of predictive coding—associate attentional gain with the expected precision of sensory information. Formal models of the Posner paradigm suggest that validity effects can be explained in a principled (Bayes optimal) fashion in terms of a cue-dependent setting of precision or gain on the sensory channels reporting anticipated target locations, which is updated selectively by invalid targets. This normative model is equipped with a biologically plausible process theory in the form of predictive coding, where precision is encoded by the gain of superficial pyramidal cells reporting prediction error. We used dynamic causal modeling to assess the evidence in magnetoencephalographic responses for cue-dependent and top-down updating of superficial pyramidal cell gain. Bayesian model comparison suggested that it is almost certain that differences in superficial pyramidal cells gain—and its top-down modulation—contribute to observed responses; and we could be more than 80% certain that anticipatory effects on post-synaptic gain are limited to visual (extrastriate) sources. These empirical results speak to the role of attention in optimizing perceptual inference and its formulation in terms of predictive coding.

## Introduction

Several years ago, we suggested that attention can be understood as the selection of processing channels that conveyed precise or salient information within the framework of predictive coding (Feldman and Friston, 2010). The idea is that both the content of visual information and the confidence placed in that information have to be inferred during perception. In predictive coding, top-down predictions of the content are confirmed or disconfirmed by comparison with bottom-up sensory information (Rao and Ballard, 1999; Friston, 2005). However, this comparison rests on estimating the reliability or precision of sensory information—or more exactly the residuals or prediction error that cannot be explained. This precision may be itself context sensitive and has to be updated in exactly the same way as predictions of content (Brown and Friston, 2012a,b). This leads to view of hierarchical perceptual synthesis in which particular processing channels are selected on the basis of cues that portend spatial locations or featural attributes that are likely to convey precise information. In neuronally plausible implementations of this hierarchical Bayesian inference—namely, generalized Bayesian filtering or predictive coding—expected precision is thought to be encoded by the post-synaptic sensitivity or gain of cells reporting prediction error (Friston and Kiebel, 2009). Given that prediction error is passed forward from sensory cortex to higher cortical areas by ascending or forward connections, the most likely candidates for reporting prediction error are the superficial pyramidal cells that are the source of ascending connections (Bastos et al., 2012). This means that one can understand attention as the top-down gain control of superficial pyramidal cells passing information that is yet to be explained (i.e., prediction error) deep into the visual hierarchy.

This normative model and its neuronal implementation have been used to simulate and reproduce both the psychophysical and electrophysiological characteristics of the Posner paradigm (Feldman and Friston, 2010). In brief, predictive cues engage top-down predictions of increased precision in the left or right hemifield that facilitate the rapid processing of (inference about) valid visual targets. However, when an invalid target is presented in the wrong hemifield, the evidence accumulation implicit in predictive coding is slower, because gain or precision acts as a synaptic rate constant. This leads to protracted reaction times and an invalidity cost. Simultaneously, the scheme infers that prior beliefs about the target have been violated and prediction errors drive higher levels to update both the deployment of attention (i.e., precision) and target predictions *per se*. This explains the classic electrophysiological correlates of the validity effects in the Posner paradigm—in which invalid targets elicit slightly attenuated P1, N1 and N2 early components and a more pronounced P3b late component (Mangun and Hillyard, 1991; Hugdahl and Nordby, 1994; Talsma et al., 2007). These two electrophysiological characteristics may reflect the initial insensitivity (low precision or gain) of early visual responses and a subsequent *post-hoc* revision of top-down precision or gain control, when prediction error cannot be resolved by predictions based upon the (invalid) cue.

In this paper, we tried to verify these explanations for electromagnetic responses to valid and invalid targets in the Posner paradigm using magnetoencephalography (MEG) and dynamic causal modeling of differences in effective connectivity. In particular, we hoped to establish that a sufficient explanation for responses evoked by valid and invalid targets would be provided by a difference in the gain or post-synaptic sensitivity of superficial parietal cells following a cue—and a subsequent top-down modulation of this gain from parietal and higher extrastriate sources. To do this, we needed to use dynamic causal models based on canonical microcircuits that distinguish between superficial and deep pyramidal cells (Bastos et al., 2012)—and that explicitly include a top-down modulation of superficial pyramidal cells.

In what follows, we provide a brief description of the dynamic causal models used to address precision or gain control in predictive coding; describe the data and experimental design; and report the results of Bayesian model comparisons that quantify the evidence for condition-specific differences in superficial pyramidal cell gain. Our focus here is on cue-dependent differences in gain prior to the onset of a visual target and subsequent top-down modulation of that gain during target processing. In particular, we asked whether cue-dependent differences in gain, top-down modulation or both were evident in evoked electromagnetic responses—and, whether any differences in gain were restricted to visual sources or extended to the parietal cortex.

## Materials and methods

### Dynamic causal modeling of predictive coding

**Figure 1:**
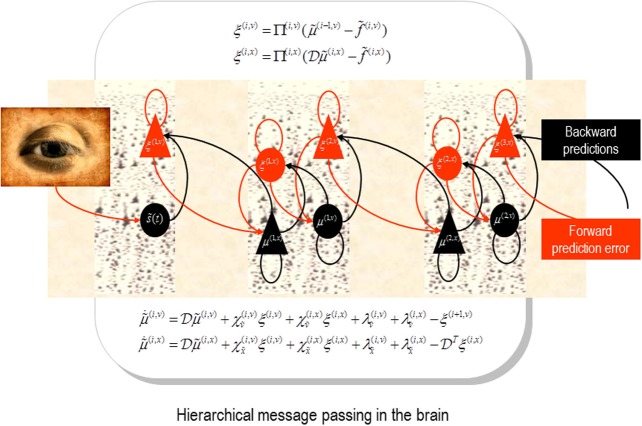
**Schematic detailing the neuronal architecture that might implement generalized predictive coding**. This shows the speculative cells of origin of forward driving connections that convey prediction error from a lower area to a higher area and the backward connections that construct predictions (Mumford, 1992; Friston et al., 2006). These predictions try to explain away prediction error in lower levels. In this scheme, the sources of forward and backward connections are superficial and deep pyramidal cells respectively. The equations represent a gradient descent on free-energy under a hierarchical dynamic model (see Feldman and Friston, 2010). State-units are in black and error-units in red. Here, neuronal populations are deployed hierarchically within three cortical areas (or macro-columns). Subscripts denote derivatives.

In predictive coding models of inference in the brain (Mumford, 1992; Friston, 2005; Bastos et al., 2012), prediction error ascends to update representations at higher hierarchical levels. See Figure 1 for a schematic summary. Crucially, the excitability of cells reporting prediction error corresponds (mathematically) to the precision of—or confidence in—the information they convey. This precision has been used to explain the psychophysical and electrophysiological correlates of attention and can be regarded as the basis of selective (predictive or attentional) gain—in which sensory processing channels that convey precise information are enabled.

Neurobiological implementations of predictive coding use superficial pyramidal cells to report precision-weighted prediction error: $\xi^{\left(i\right)}=\Pi^{\left(i\right)}\cdot ({\tilde{\mu }}^{\left(i\right)}-f({\tilde{\mu }}^{\left(i+1\right)}))$, where ${\tilde{\mu }}^{\left(i\right)}$ corresponds to representations (posterior expectations) of states of the world at level *i* in a cortical hierarchy and $f({\tilde{\mu }}^{\left(i+1\right)})$ corresponds to the top-down predictions of these expectations—based upon expectations in the level above. The precision of the ensuing prediction error is modulated by the precision Π^(*i*)^ to weight prediction errors in proportion to their (expected) reliability (c.f., known uncertainty). From our point of view, the encoding of precision—at each level of the hierarchy—can be associated with the strength of inhibitory recurrent connections by noting that the expression for prediction errors is the solution to the following equation describing neuronal dynamics.

$$\begin{array}{l} {\dot{\xi }}^{\left(i\right)}={\tilde{\mu }}^{\left(i\right)}-f({\tilde{\mu }}^{\left(i+1\right)})-\exp(\gamma^{\left(i\right)})\cdot \xi^{\left(i\right)}\\ {\dot{\xi }}^{\left(i\right)}=0\Rightarrow \gamma^{\left(i\right)}=-\text{In}\Pi \end{array}$$

A more complete exposition of these dynamics can be found in Friston (2005). In this equation, γ^(*i*)^ is the negative log precision.

With Dynamic Causal Modeling (Garrido et al., 2008; Bastos et al., 2012), we map this neurobiological implementation of predictive coding onto a neural mass model which is capable of simulating MEG data. The depolarization of the three excitatory cell populations in the model—superficial and deep pyramidal cells, as well as spiny stellate cells, forms the output of the model with the main contribution coming from superficial pyramidal cells. This activity is transformed by an MEG-specific lead-field which describes the translation from source activity to sensor perturbation.

The four-population neural mass model used here has been described before (Brown and Friston, 2012b). In the neural mass models, γ^(*i*)^, the negative log precision, corresponds to the strength of recurrent inhibitory connections on superficial pyramidal cells. This means that as preclon increases, the strength of recurrent inhibition decreases. We therefore use the strength of intrinsic (recurrent) self-inhibition (on superficial pyramidal cells) as a proxy for log precision.

One new feature is introduced in this implementation of the neural mass model. To model top-down modulation of this self-inhibition we use the following form of (backward) modulatory connectivity:

$$\gamma^{\left(i\right)}=\gamma_{0}^{\left(i\right)}-32\;\cdot \;M\;\cdot \;\left(\sigma (V)-\sigma_{0}\right)$$

Here, γ_0_ is self-inhibition when firing rates are at baseline levels σ_0_ = σ(0). Firing rates σ(*V*) ∈ [0, 1] are a sigmoid function of depolarization *V* ∈ ℝ of afferent neuronal populations (deep pyramidal cells in other sources). The modulatory connection strength matrix *M*weights the influence of other sources; such that a high value suppresses self-inhibition and (effectively) increases the gain or precision of the superficial pyramidal cells that are targeted.In what follows, we will model condition (valid or invalid) specific effects on γ to evaluate the evidence for cue-dependent changes in gain at the onset of target processing and test for condition specific changes in *M* that mediate target-dependent changes in gain as target is processed. Our hope was that we will find evidence for differences in baseline gain and subsequent top-down modulation—and that these would be expressed predominantly in early visual sources.

Specifically, we anticipated that intrinsic self-inhibition would be lower (gain would be higher) in left hemisphere sources after (invalid) cueing of the right hemifield relative to (valid) cueing of the left hemifield, where the target appeared in the left hemifield in both conditions. In other words, we hoped to show differential responses to identical targets could be explained by differences in gain induced by valid and invalid cues. Furthermore, we anticipated differences in descending modulatory effects between valid and invalid trials that would be necessary to reverse the laterality of gain control following an invalid target.

### Participants

Fourteen healthy right-handed subjects participated in the study (8 male; age 20–54). Ethical approval was obtained from the UCL Research Ethics Committee (no. 2715/001). Written informed consent was obtained from all subjects.

### Experimental paradigm

All stimuli were presented using Matlab 7.1 and Cogent (http://www.vislab.ucl.ac.uk/cogent.php). Stimuli were projected onto a screen 70 cm from the subjects. During the task, subjects fixated on a central cross at all times. At the start of each trial, the cross was replaced by an arrow pointing to the bottom left or bottom right corner of the screen, or a double-headed arrow pointing to both (neutral trials). The cues subtended 1.6 degrees of visual angle. After a cue-target interval of 50, 100, 200, or 400 ms, a target appeared either where the arrow had indicated (valid) or at the other side (invalid). The target was a white circle subtending 3.1 degrees of visual angle and presented in the lower left or lower right corners of the screen at 14.7 degrees eccentricity. Participants pressed a button with their right hand as soon as the target appeared. 66% of trials were valid, 17% were invalid and 17% uninformative (neutral cue trials are not considered here). Left and right cues and targets were balanced. Catch trials, in which no target followed the cue, were randomly presented before 10% of trials. 1800 trials were collected over three sessions on two consecutive days.

### Behavioral data

Reaction times were collected by Cogent and analyzed with IBM SPSS 20. A full factorial univariate ANOVA was performed with fixed factors “side” “validity” and “cue-target interval” and random factor “subject.”

### Data collection and processing

MEG data was obtained using a whole-head 275-channel axial gradiometer MEG system (CTF Systems). The sampling rate was 600 Hz and a low-pass filter of 150 Hz was applied. Head position was monitored using three localization coils, placed on the nasion and in front of each ear. An infrared eyetracker (Eyelink 1000) was used to monitor participants' fixation as well as to detect blinks. Stimuli were presented and behavioral data were collected with Cogent.

Data were analyzed using SPM12b for EEG/MEG. Data were down-sampled to 200 Hz and bandpass-filtered between 2 Hz and 32 Hz. Baseline-corrected epochs were extracted from the time series starting at 50 ms before target onset and ending 400 ms after target onset. Trials where the eyetracker detected a blink or saccade were excluded from analysis. Trials were then robustly averaged across cue-target intervals and participants to yield four conditions—left valid cue, right valid cue left invalid cue and right invalid cue. Averaging across participants can reduce the spatial precision of the MEG signal; however, as our hypotheses were not concerned with the spatial location of the signals we chose to combine data across all participants to increase the signal-to-noise ratio of the waveforms.

### Data feature and source specification

We addressed our hypothesis using condition-specific grand average responses over all subjects. Intuitively, this is like treating each subject as if they were the same subject to produce an average ERP. To identify plausible sources we used a distributed source reconstruction (using four grand averages: valid right target, invalid right target, valid left target, and invalid left target) based on multiple sparse priors (with default settings).

**Figure 2:**
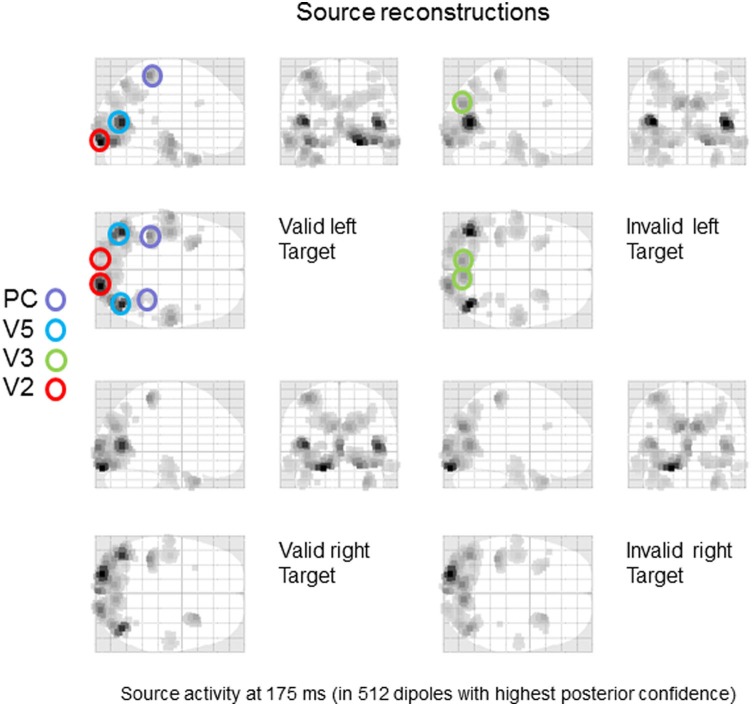
**Source specification for dynamic causal modeling**. A distributed source reconstruction was performed (Mattout et al., 2006) and the power of evoked responses was quantified over the time course of the trial and all frequencies to yield the maximum intensity projections shown. Eight sources corresponding (roughly) to key maxima of source activity were identified: included bilateral early visual sources (V2); bilateral sources near the occipitotemporal-parietal junction (V5); bilateral dorsal (V3) extrastriate sources and bilateral superior parietal sources (PC).

The grand average data were bandpass filtered between 2 and 32 Hz and windowed from 0–400 ms of peristimulus time. We used a lead field based upon the standard MRI template and a boundary element model as implemented in SPM12 (Mattout et al., 2006). After source reconstruction, we quantified the power of evoked responses (over all frequencies and peristimulus time) to produce the maximum intensity projections in Figure 2. As one would expect, left targets activate right early visual sources and *vice versa*. Note further, that early visual source responses to valid left targets are greater than the same targets under invalid cues. On the basis of these reconstructions, we identified eight sources corresponding (roughly) to key maxima of source activity. These sources included bilateral early visual sources (V2); bilateral sources near the occipitotemporal-parietal junction (V5); bilateral dorsal (V3) extrastriate sources and bilateral superior parietal sources (PC). The anatomical designation of these sources should not be taken too seriously—they are used largely an aide-memoire for sources at various levels in the visual hierarchy, so that we can discuss the functional anatomy. Clearly, the spatial precision of source localization does not allow us to associate each source with a specific cytoarchitectonic area—and even if we could, there is sufficient intersubject variability in cortical architectures to make this association, at best, heuristic.

**Figure 3:**
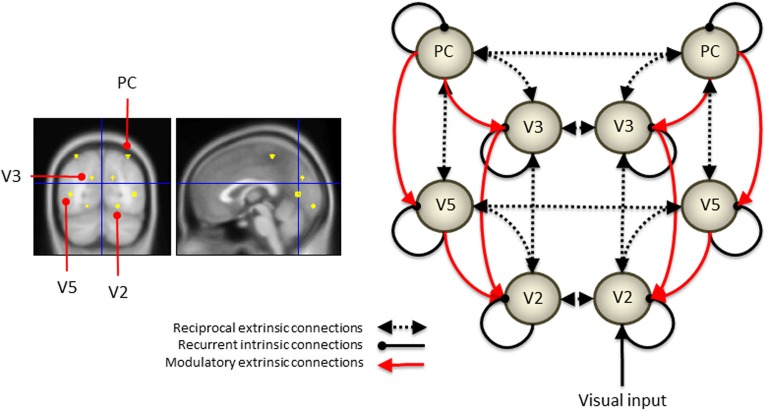
**The location of the eight sources is shown in the panels on the left**. To construct the DCM, these sources were connected in the distributed network shown on the right. The parietal sources sent both driving and modulatory backward connections to the extrastriate (V3 and V5) sources that then sent backward connections to the V2 sources. These connections were reciprocated by extrinsic forward connections to produce a simple visual hierarchy with bilateral connections.

The distributed network constituting the DCM is shown in Figure 3. The parietal sources sent backward connections to the extrastriate (V3 and V5) sources that then sent backward connections to the V2 sources. These connections were reciprocated by extrinsic forward connections to produce a simple visual hierarchy with bilateral connections.

### Model space and Bayesian model comparison

The DCM analyses used data from 0 to 400 ms of peristimulus time. To de-noise the data and improve computational efficiency, we fitted the first eight canonical modes of the scalp data, given the source locations—these can be regarded as the principal components of the data that can be explained by source activity. The sources were modeled as small cortical patches of about 16 mm radius—centered on the source locations in Figure 2—as described in (Daunizeau et al., 2006). The vertices of these sources used the same lead fields as in the source reconstruction.

Exogenous (visual target related) input was modeled as a Gaussian function with a prior peak at 120 ms (and a prior standard deviation of 16 ms). This input was delivered to V2 on the appropriate side (left for right target trials and right for left target trials). The ensuing models were optimized to explain sensor responses by adjusting their (neuronal and lead field) parameters in the usual way—this is known as model inversion or fitting. The products of this inversion are posterior estimates of (differences in) intrinsic and extrinsic connectivity and the evidence or marginal likelihood for each model considered.

Our hypothesis centered on the gain of superficial pyramidal cells. We therefore estimated a full model in which all intrinsic gains and their extrinsic (backward) modulation could differ between valid and invalid trials. To ensure the same stimuli were used for assessing these differences we conducted two sets of analyses—one for targets presented to the left visual field and another for targets presented on the right. Each DCM estimated all intrinsic, extrinsic and modulatory connection strengths and any differences in intrinsic and modulatory connections due to invalid cuing.

After inverting the full model we then evaluated the evidence for reduced versions that constitute alternative hypothesizes or models. This model space was created by partitioning connectivity differences into three subsets and considering all eight combinations. These subsets were changes in intrinsic gain in the extrastriate sources (V2, V3, and V5); changes in parietal (PC) gain and changes in extrinsic modulatory connections. This partition was motivated by distinguishing between the effect of the cue on target-related responses—which should be apparent in changes in intrinsic gain in the visual areas—and the effect of the target *per se*—which should be apparent in changes in backward modulation of gain. To evaluate the ensuing models, we use Bayesian model comparison based upon (a variational free energy) approximation to log evidence. Having identified the model with the greatest evidence, we then examined its posterior parameter estimates. This allowed us to characterize validity effects quantitatively and to interpret them in computational (predictive coding) terms.

## Results

### Behavioral data

**Figure 4:**
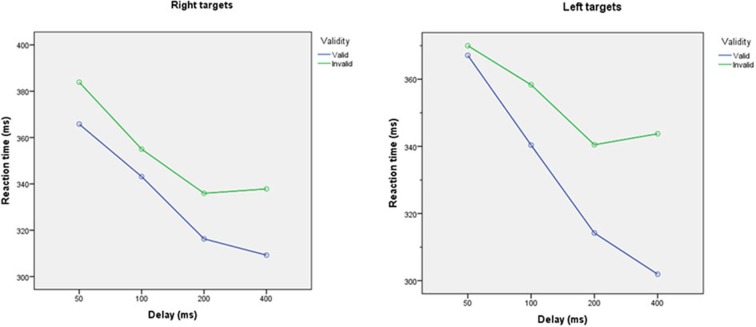
**Reaction times to validly and invalidly cued targets at different cue-target intervals for targets appearing on the left (left panel) and right (right panel), averaged across all participants**. Reaction times were faster for validly than invalidly cued targets (*p* < 0.0001). Reaction times decreased as cue-target interval increase (all *p* < 0.05).

The ANOVA demonstratated significant main effects of validity, subject and cue-target interval, with significant interactions between cue-target interval∗validity, cue-target interval∗subject, side∗cue-target interval and validity∗side∗subject. Reaction times to validly cued targets were significantly shorter than to invalidly cued targets [left: mean (SD) 333 ms (42 ms) vs. 355 ms (44 ms), *p* < 0.001; right: mean (SD) 334 ms (42 ms) vs. 354 ms (44 ms)], Figure 4.

### Attentional effects in sensor space

The effects of attention (validity of cueing) on responses to targets presented in the left hemifield are shown—for the first two canonical modes—in Figure 6. Although these MEG responses are formally distinct from classic EEG results, they speak to similar effects on early and late responses: the blue lines correspond to valid trials and red lines to invalid trials. The response in the first mode shows the early response (just before 200 ms) has a reduced latency and slightly higher amplitude—consistent with an attenuation of N2 response to invalid targets, as seen in classic EEG studies (Mangun and Hillyard, 1991). In terms of late responses, the second mode shows a protracted and elevated response around 300 ms that is consistent with a P3b component, when the target location is not attended.

The solid lines report the model predictions of observed responses (broken lines) in sensor space after inversion of the DCM. These illustrate the accuracy of model inversion, capturing both the early and late differences to a considerable level of detail. Examples of the underlying source activity that generates these predictions are shown in the lower panel. These traces represent the depolarization of three excitatory populations within the left V2 source, contralateral to the visual input modeling the effects of target presentation. The dotted lines correspond to the spiny stellate and deep pyramidal populations, while the solid lines report the superficial pyramidal cells—that are the predominant contributors to sensor data. Note that this level of reconstructed neurophysiological detail rests on having a biologically plausible forward model.

Somewhat to our surprise, the differential responses to right targets were much less marked (results not shown). Furthermore, model inversion failed to converge for these conditions. Therefore, we restricted our analysis to the left target conditions. The failure to elicit clear validity effects with right targets may relate to the asymmetry of responses—and attentional gain control (see below).

### Bayesian model selection

**Figure 5:**
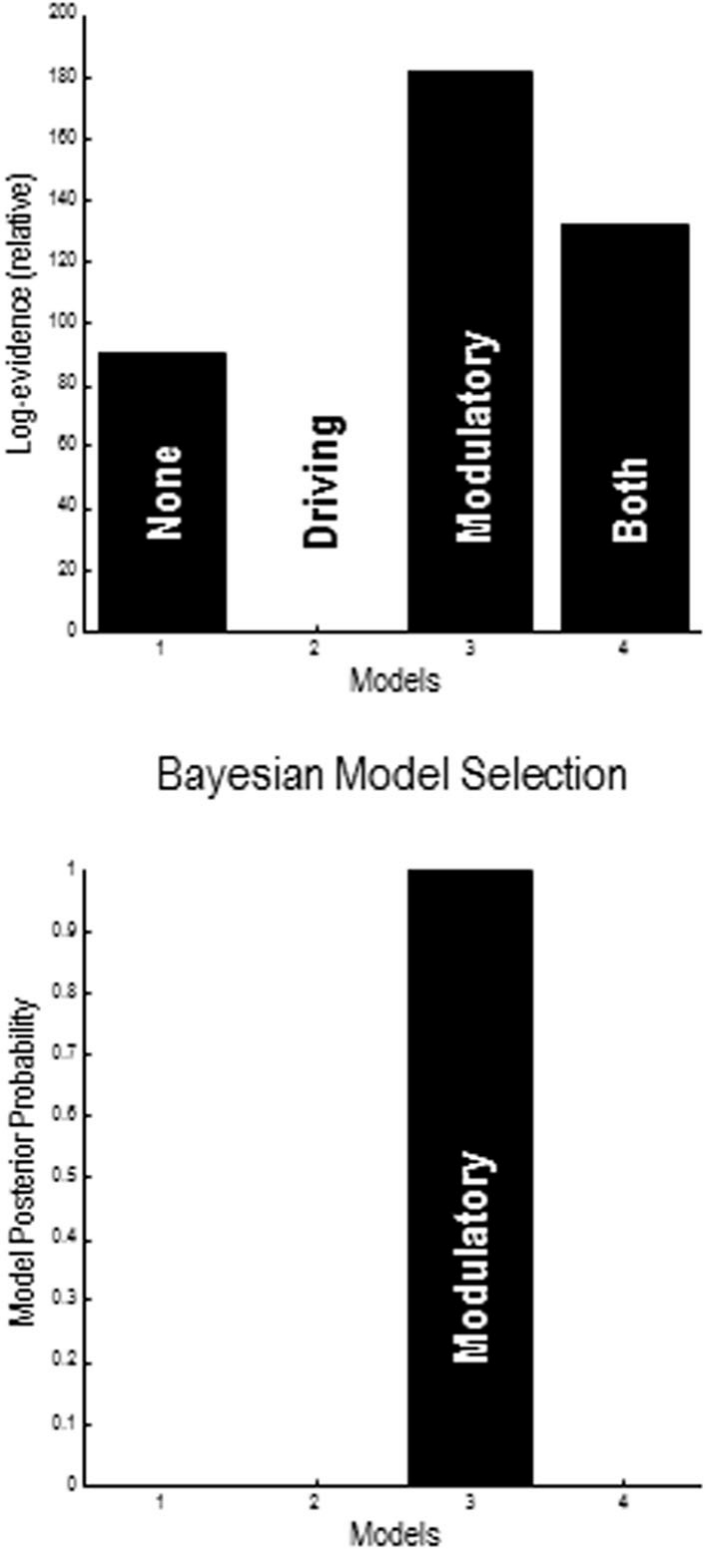
**Results of provisional Bayesian model selection**. The (free energy approximation) to log evidence was assessed for models with and without validity–dependent differences in top-down driving and modulatory connections. The log evidences **(upper panel)** show that the model with differences in modulatory connections has the greatest posterior probability **(lower panel)**. The log evidences are shown relative to the evidence for a null model with no changes in either driving or modulatory backward connections.

A provisional Bayesian Model Comparison demonstrated that modeling the validity effect with changes in the strengths of the modulatory backwards connections only had the greatest posterior probability, justifying the investigation of these connections in the following analyses (Figure 5). The comparison of different explanations for the validity effects above focused on differences in the gain of superficial pyramidal cells—either intrinsic to extrastriate or parietal sources, or differences in the modulation of gain, mediated by extrinsic top-down connections. The relative log evidences for all combinations of these condition-specific differences are shown in the upper left panel of Figure 7. The labeling of these models indicates the presence or absence of differences in extrastriate gain, parietal gain and gain modulation. It can be seen that the model with the greatest evidence includes differences in extrastriate gain and gain modulation—but not differences in parietal gain. The corresponding posterior probabilities of these models (assuming all were equally plausible *a priori*) are shown in the upper right panel. These suggest that we cannot definitively exclude differences in parietal gain; however, we can be more than 80% confident that parietal effects are not necessary to explain these data, provided we allow for validity effects on extrastriate gain and its top-down modulation.

**Figure 6:**
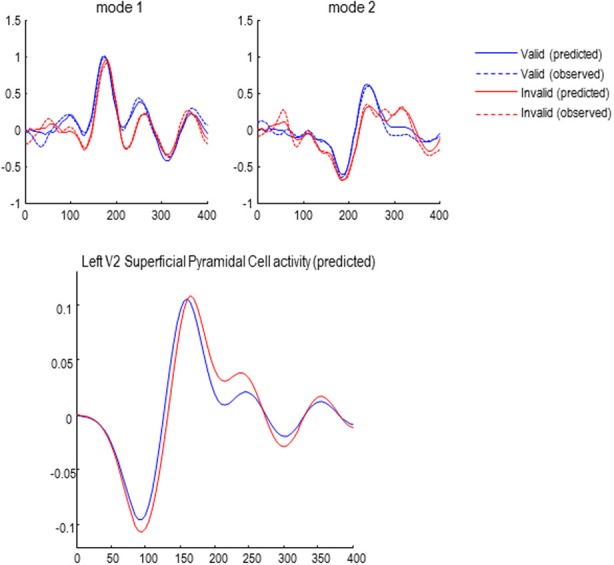
**Upper panel:** the first two of eight spatial modes (principle components) of the data to which the DCMs were fitted. Observed responses are dashed lines; solid lines show the responses fitted by the winning model (see below), demonstrating a good model fit. **Lower panel:** reconstructed source activity in left V2.

**Figure 7:**
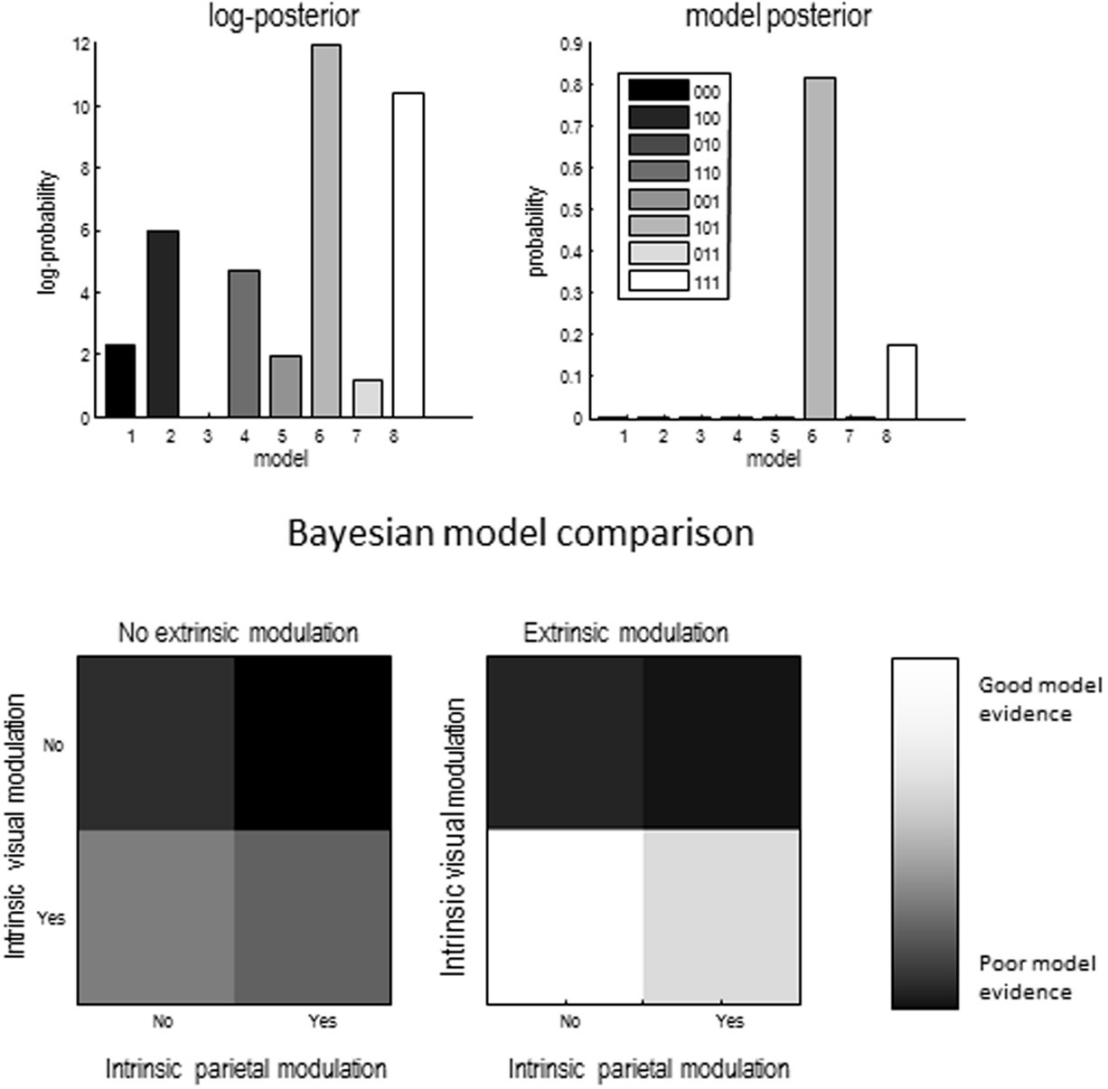
**Upper left panel:** relative log evidence for models which fitted differences between conditions through changes in one of three sets of parameters: superficial pyramidal cell gain in visual areas (1 _ _), superficial pyramidal cell gain in parietal areas (_ 1 _) and strength of backwards modulatory connections (_ _ 1). **Upper right panel:** The winning model had changes in superficial pyramidal cell gain in visual areas and in the strength of backwards modulatory connections, meaning that we can be more than 80% certain that backwards modulatory connections are not necessary explain the electrophysiological signatures of the validity effect. **Lower panels** show the same data as in the **top left panel**, but in image format.

The lower panels show the same log evidences but in image format, to illustrate the relative evidence for gain effects. The image on the right is under extrinsic top-down gain modulation and suggests greater evidence than the corresponding results on the left, where modulatory effects are concluded. In both cases, the model with extrastriate—but not parietal—gain differences has the greatest evidence. Having identified the best model, we then quantified the changes in model parameters that explain the validity effect.

### Attentional gain effects

**Figure 8:**
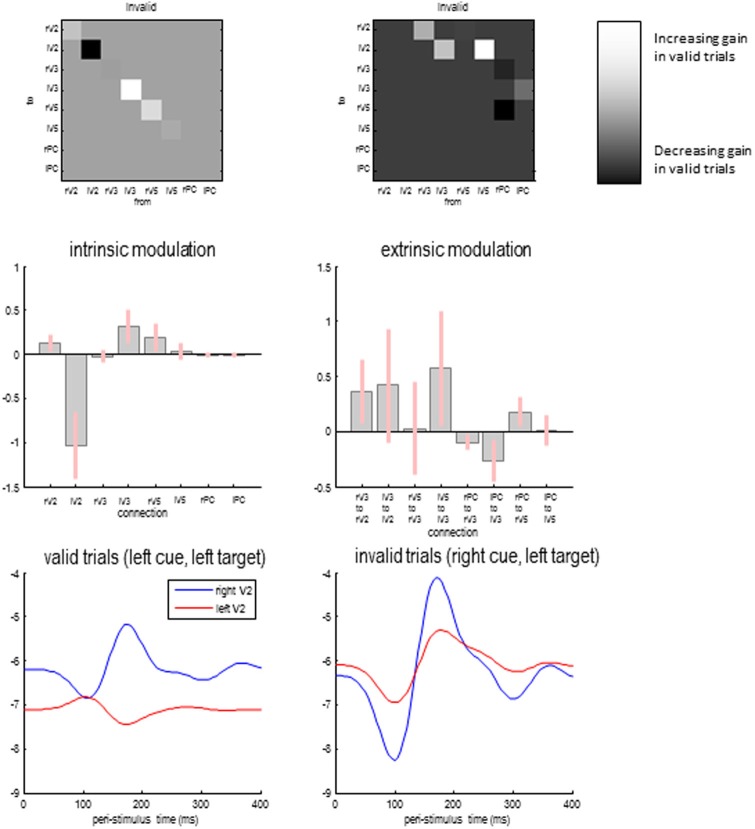
**Differences in self-inhibition (upper left panels) and backwards modulation of self-inhibition (upper right panels) between valid and invalid trials for the model with the highest posterior probability above**. The **lower panels** show the gain of the superficial pyramidal cells over time in valid and invalid trials.

Figure 8 shows the differences in self-inhibition (top left panels) and backwards modulation of self-inhibition (top right panels) for the model with the highest posterior probability above. The upper panels show the differences as connectivity matrices indicating changes in connection strength. This means that differences in self-inhibition are located along the leading diagonal, while differences in backward connections are restricted to the upper diagonal elements. The middle panels show the same results but in terms of the posterior expectations for differences (in connections that changed) and their 90% confidence intervals.

As anticipated, the recurrent or self-inhibition of early visual sources showed a highly asymmetrical difference when attending to the right hemifield (during invalid trials), compared to attending to the left hemifield (during valid trials). When attending to the right hemifield the left V2 source shows a profound decrease in the self-inhibition of superficial pyramidal cells—consistent with a disinhibition or increase in gain. This is accompanied by a slight decrease in the gain or sensitivity of the left extrastriate V3 source and an increase in the right V5 source. Note that these gain differences are in place before the target is presented and—presumably—are instantiated by the cue. When the target arrives, it evokes responses throughout the visual hierarchy that modulate the gain of the lower sources. These effects are mediated by the backward modulatory connections.

With the exception of backward connections from the right parietal source, all the differences in backward modulation between valid and invalid trials are positive, speaking to an increase in gain (or a top-down disinhibition of superficial pyramidal populations). However, it is difficult to predict the changes in gain that are produced by modulatory effects, because this disinhibition could itself be inhibited when top-down afference falls below baseline firing rates. Therefore, we evaluated the changes in gain in early visual sources as a function of peristimulus time for the two conditions. This is possible because we have a biologically plausible forward or generative model that allows us to examine changes in both neuronal states and connectivity—over peristimulus time—using the posterior parameter estimates.

Figure 8 shows the log gain or precision of the early visual sources, following target presentation for valid (lower left panel) and invalid trials (lower right panel). As expected, there is a marked asymmetry in gain modulation during the prestimulus period that is revised or updated after the target is processed—through activity dependent modulatory mechanisms. Specifically, during valid trials the gain is greater in the appropriate (right) early visual source and then reaches a peak shortly before 200 ms. This peak is complemented by a suppression of gain in the unattended (left) visual source. This can be contrasted with the gain modulation during invalid trials. Here, the attended left source starts off with a slightly higher gain. Furthermore, the unattended source is suppressed more acutely with the arrival of the target. However, after about 120 ms its gain increases markedly, to peak just before 200 ms. This redeployment of precision (c.f., reorientation of attention) is the largest gain modulation in both sources and conditions. Interestingly, the gain of the left source also enjoys a slight increase but to a substantially lesser degree. In short, the top-down modulation of gain (through modulatory disinhibition of superficial pyramidal cells) appears to exert a dynamic gain control over peristimulus time and shows marked lateralization, when attention is switched from one hemifield to another.

## Discussion

In conclusion, we have used dynamic causal modeling to characterize putative changes in the gain of superficial pyramidal cell populations that might underlie attentional (validity) effects in the Posner paradigm. Our focus on gain mechanisms was motivated by theoretical formulations of attention in terms of optimizing perceptual inference using the expected precision of particular processing streams (Feldman and Friston, 2010). This formulation rests upon predictive coding schemes that the brain might use to infer the causes of sensory consequences it has to explain (Friston and Kiebel, 2009). Our model comparison and quantitative analysis of changes in parameter estimates are remarkably consistent with theoretical predictions.

In brief, the modeling results suggest that, following a cue, sensory channels in the appropriate hemisphere are afforded more precision through the disinhibition of recurrent or self-inhibition of superficial pyramidal cells. These cells are thought to pass sensory information (prediction error) to higher levels to inform perception. When a target appears in an unattended location, the misplaced gain or sensitivity of lower areas is revised or updated by top-down modulatory influences from higher extrastriate and parietal sources. Phenomenologically, this increases the latency and reduces the amplitude of early responses to invalid targets—because they are processed by channels that have an inappropriately low gain. The resulting prediction error induces an update response that reverses the misattribution of gain, producing differences in late or endogenous response components—such as the P3b. The P3b is known to be sensitive to probabilistic surprise (Mars et al., 2008; Kolossa et al., 2012) as well as to risk (Schuermann et al., 2012). These results suggest that the larger P300 in response to more unexpected events might be a result of exaggerated precision at lower levels incited by the arrival of an unexpected stimulus.

This application of dynamic causal modeling is slightly more focused than normal applications. We did not explore a large model space but focused on particular synaptic mechanisms as sufficient explanations for condition-specific responses. It is more than likely that there are many models of these differential responses that would produce equally good or better explanations. However, we chose to focus on models that were explicitly informed or constrained by computational and biophysical considerations; namely, that the effects have to be mediated by a neurobiologically plausible gain control that is consistent with normative principles of perceptual inference. This allowed us to validate the theoretical proposals empirically, while providing a principled model space within which to test specific hypotheses about the underlying wetware.

Evidence suggests that gain modulation in pyramidal cells is an important mechanism in visual attention. Electrophysiological studies have demonstrated that attention can enhance the response of visual neurons (likely to be pyramidal cells) by a multiplicative factor (McAdams and Maunsell, 1999; Treue and Martínez-Trujillo, 1999). fMRI studies demonstrate increased BOLD response for attended versus unattended stimuli (Kastner et al., 1998), even if these stimuli are predictable (Kok et al., 2012), and early visual ERPs, which are most strongly determined by pyramidal cell firing, are enhanced by attention (Rauss et al., 2011).

Interestingly, although we were almost forced to model gain control using inhibitory self connections—because of the relative simplicity of neuronal mass models used by dynamic causal modeling—this particular mechanism makes a lot of sense in relation to current thinking about attention. Convergent evidence implicates local inhibitory processing, mediated by GABAergic neurotransmission, in attention. Drugs working at GABA receptors, such as benzodiazepines, which are positive allosteric modulators of GABA-A receptors, increase the behavioral effect of cues so that reaction time differences to validly and invalidly cued targets become larger, while overall reaction times are slowed (Johnson et al., 1995). Nicotine (an agonist at nicotinic acetylcholine receptors) also affects reaction times in the Posner paradigm, but it decreases the validity effect while increasing reaction times (Thiel et al., 2005; Meinke et al., 2006), and it is believed that the attentional effects of acetylcholine might be mediated at least partly though depression of inhibitory interneuron activity (Xiang et al., 1998; Buia and Tiesinga, 2006). These contrasting effects suggest that the inhibitory interneurons set the gain of their cortical area to determine reaction times. Increasing their effects increases reaction times due to greater overall inhibition, exaggerating the difference between high- and low-gain cortical areas, and *vice versa*. This is consistent with the “biased activation theory” of selective attention (Grabenhorst and Rolls, 2010), which suggests that GABA interneurons mediate competition between stimuli which can be biased through top-down signals (the backwards modulatory connections in this DCM).

In summary, the emerging picture is that attention may be mediated through local intrinsic or recurrent inhibitory mechanisms that form a key part of cortical gain control—and that have characteristic signatures in terms of frequency specific induced responses. This fits comfortably with the theoretical perspective provided by predictive coding—that provides a computational role for recurrent inhibition in encoding the gain or precision of prediction errors in hierarchical processing. The results presented in this paper provide an initial link between these computational imperatives and plausible mechanisms at the level of synaptic processing and hierarchical neuronal circuits.

### Conflict of interest statement

The authors declare that the research was conducted in the absence of any commercial or financial relationships that could be construed as a potential conflict of interest.
